# Rapid antibacterial activity of anodized aluminum-based materials impregnated with quaternary ammonium compounds for high-touch surfaces to limit transmission of pathogenic bacteria†

**DOI:** 10.1039/d1ra07159a

**Published:** 2021-11-26

**Authors:** Jessica Jann, Olivier Drevelle, X. Grant Chen, Myriam Auclair-Gilbert, Gervais Soucy, Nathalie Faucheux, Louis-Charles Fortier

**Affiliations:** Department of Chemical and Biotechnological Engineering, Faculty of Engineering, Université de Sherbrooke 2500 boul. de l'Université Sherbrooke Québec J1K 2R1 Canada Nathalie.Faucheux@USherbrooke.ca; Clinical Research Center of Centre Hospitalier Universitaire de Sherbrooke 12e Avenue N Sherbrooke Québec J1H 5N4 Canada; Department of Applied Science, University of Quebec in Chicoutimi Saguenay Quebec G7H 2B1 Canada; A3 Surfaces 1433, Rue de la Manic Chicoutimi Québec G7K 1G8 Canada; Department of Microbiology and Infectious Diseases, Faculty of Medicine and Health Sciences, Université de Sherbrooke 3201 rue Jean Mignault Sherbrooke Québec J1E 4K8 Canada Louis-Charles.Fortier@USherbrooke.ca

## Abstract

Infections caused by multidrug-resistant bacteria are a major public health problem. Their transmission is strongly linked to cross contamination *via* inert surfaces, which can serve as reservoirs for pathogenic microorganisms. To address this problem, antibacterial materials applied to high-touch surfaces have been developed. However, reaching a rapid and lasting effectiveness under real life conditions of use remains challenging. In the present paper, hard-anodized aluminum (AA) materials impregnated with antibacterial agents (quaternary ammonium compounds (QACs) and/or nitrate silver (AgNO_3_)) were prepared and characterized. The thickness of the anodized layer was about 50 μm with pore diameter of 70 nm. AA with QACs and/or AgNO_3_ had a water contact angle varying between 45 and 70°. The antibacterial activity of the materials was determined under different experimental settings to better mimic their use, and included liquid, humid, and dry conditions. AA–QAC surfaces demonstrated excellent efficiency, killing >99.9% of bacteria in 5 min on a wide range of Gram-positive (*Staphylococcus aureus*, *Clostridioides difficile*, vancomycin-resistant *Enterococcus faecium*) and Gram-negative (streptomycin-resistant *Salmonella typhimurium* and encapsulated *Klebsiella pneumoniae*) pathogens. AA–QACs showed a faster antibacterial activity (from 0.25 to 5 min) compared with antibacterial copper used as a reference (from 15 min to more than 1 h). We show that to maintain their high performance, AA–QACs should be used in low humidity environments and should be cleaned with solutions composed of QACs. Altogether, AA–QAC materials constitute promising candidates to prevent the transmission of pathogenic bacteria on high-touch surfaces.

## Abstract 2

Anodized aluminum-based antibacterial materials impregnated with biocidal compounds for high-touch surfaces applications to limit transmission of pathogenic bacteria.

## Graphical abstract

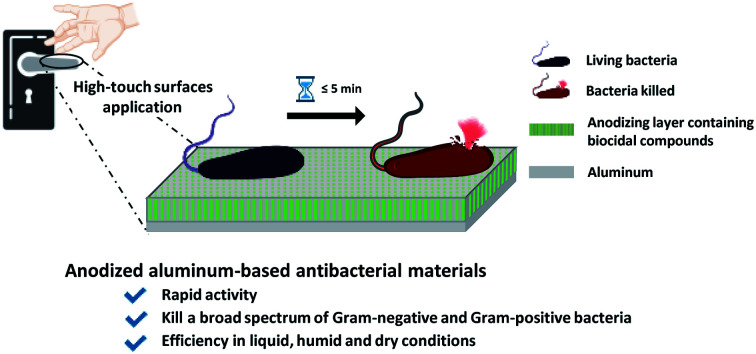

## 1. Introduction

Bacterial nosocomial infections represent an extremely serious global threat to human health.^1–3^ Drug-resistant infections are currently responsible for 700 000 deaths per year worldwide and if the current situation does not improve, this human toll might reach 10 million deaths per year by 2050.^3^ The direct (healthcare) and indirect (international trade, animal production) impacts of these infections also place a heavy financial burden on global systems, estimated between 1.0 and 3.4 $ billion per year.^4,5^ Finding new drugs to fight bacterial pathogens is urgent but developing new strategies to break the transmission chain and prevent propagation of these bacteria is also crucial.

The transmission of bacterial pathogens that cause infections is mainly linked to cross-contamination through inert surfaces and the environment.^1,6–8^ An interesting way to limit the spread of pathogenic bacteria, without the use of antibiotics, is to develop antibacterial materials for use in high contact areas, such as door handles, stair railings, components of public transport and other surfaces that can serve as reservoirs for microorganisms.^8–10^

Several strategies can be used to design antibacterial materials. One of them is to select a type of material with inherent antibacterial activity such as metal alloys, mainly based on copper, silver, or zinc.^10–13^ Copper-based alloys are currently the most used with over 500 materials registered as antibacterial products by the U.S. Environmental Protection Agency (EPA). They are capable of killing 99.9% of pathogenic bacteria within 2 h.^14–16^ However, several issues have been raised with copper-based alloys, such as durability, corrosion susceptibility, bacterial resistance selection and cost-effectiveness.^17^ In addition, their effectiveness to prevent cross-contamination or to reduce the incidence of healthcare-associated infections (HAI) was not based on blinded, randomized and unbiased clinical studies.^18–21^

Another strategy consists in applying specific coatings to acquire antibacterial surface properties (superhydrophobicity, nanostructuring) or to incorporate into the material a wide variety of known antibacterial compounds (copper, silver, quaternary ammoniums compounds (QACs), *etc.*).^10,12,22–28^ QACs are antimicrobial agents effective against a broad spectrum of Gram-positive and Gram-negative bacteria as well as several enveloped viruses, and they are widely used in cleaning products.^29,30^ The persistence of their antibacterial property over time and over a wide pH range, their odorless and amphiphilic properties, as well as the low cost of commercial grade QACs make them very good candidates for manufacturing biocidal surfaces.^31–33^ The silver ions, due to their antibacterial effect mediated by interactions and alterations of proteins and cell walls, are also used in various applications such as medical devices (central venous catheters and topical antiseptics: silver nitrate and silver sulfadiazine), textiles (clothing and bedsheets) and self-disinfecting surfaces (Surfacine™).^9,12,34,35^

Proper testing of antibacterial surfaces is critical to evaluate their effectiveness. Several international standards are currently used to assess the antibacterial properties of materials such as the Japanese standard (JIS Z2801: 2010), the European standard (ISO 22196: 2011), the American interim standard (US EPA: 2020) and its older standard more specific to copper surfaces (US EPA: 2015). These standards recommend testing antibacterial activity on model pathogens like *Staphylococcus aureus* and *Escherichia coli* (JIS Z2801 and ISO 22196) or *Pseudomonas aeruginosa* (US EPA). However, the antibacterial properties of materials are usually evaluated after long contact times such as 1–2 h to several days (*e.g.*, 99.9% of bacteria killed within 1–2 h: EPA) under liquid conditions, not representative of the practical conditions of high touch surfaces.

In the present study, we report antibacterial activity testing of anodized aluminum-based surfaces that aim to limit the spread of pathogenic bacteria.^36^ The low cost of aluminum (4 times cheaper than copper) and the anodizing process, as well as its wide range of applications (industrial, commercial and consumer goods) make anodized aluminum-based materials very interesting candidates for use in high-touch surfaces.^37^

The antibacterial materials consist of a nanoporous surface layer impregnated with antibacterial solutions. First, the aluminum-based materials were characterized after the anodization and impregnation steps by scanning electron microscopy (SEM) and contact angle measurement. The release kinetics of the impregnated compounds were also evaluated by inductively coupled plasma optical emission spectrometry (ICP-OES) and UV spectrophotometry. The antibacterial properties of the solutions selected for impregnation of the anodized aluminum (AgNO_3_ and/or QACs) were verified on Gram-positive and Gram-negative bacterial pathogens posing significant clinical problems in hospitals: *Staphylococcus aureus*, *Clostridioides difficile*, vancomycin-resistant enterococci, *Escherichia coli*, streptomycin-resistant *Salmonella* and *Klebsiella pneumoniae*. Then, the antibacterial properties of these materials were determined in a time dependent manner using experimental approaches that we developed to better mimic real life conditions of use of these materials. Copper was used as an antibacterial effectiveness control. Finally, we evaluated the antibacterial properties and tested the durability of the materials after treatment with different cleaning products and after repeated immersions into water.

## 2. Experimental section

### 2.1. Materials

#### 2.1.1. Surface materials

The materials used are hard-anodized aluminum surfaces (AA6061 and AA5052 alloys) impregnated with different antibacterial solutions: (i) AgNO_3_ (1% w/v); (ii) QACs-based (10.9% w/v) solution containing alkyldimethylbenzylammonium chloride (ADBAC), octyldecyldimethylammonium chloride, dioctyldimethylammonium chloride and didecyldimethylammonium chloride (DDAC); (iii) a combination of both AgNO_3_ (1% w/v) and QACs-based (10.9% w/v) solutions (Fig. S1†). This technology is patented by the company A3S (A3Surfaces, Chicoutimi, Quebec, Canada),^38^ who produced all anodized aluminum-based materials used in the present study. In all assays, aluminum without anodization (Al) and anodized aluminum without antibacterial solution impregnation (AA) were used as negative controls. Copper alloy C70600 (common name: CuNi10Fe1Mn) was also used as an antibacterial material control.^39^

#### 2.1.2. Bacterial strains and culture media

The selected bacterial strains which represent the vast majority of problematic nosocomial and community infections nowadays and their characteristics are listed in Table S1.†^3,4^*Staphylococcus aureus* (ATCC® 29213™) and *Escherichia coli* (ATCC® 29532™) were purchased from ATCC® (Manassas, VA, USA). *Clostridioides difficile* epidemic strain R20291 and streptomycin-resistant *Salmonella enterica* serovar *Typhimurium* SL1344 were kindly provided by Dr Trevor Lawley (Sanger Institute, United Kingdom) and the laboratory of Pr. Alfredo Menendez (Université de Sherbrooke, QC, Canada),^40^ respectively. *Klebsiella pneumoniae*, vancomycin-resistant *Enterococcus faecalis* (*Van B*) and vancomycin-resistant *Enterococcus faecium* (*Van A*) were clinical isolates obtained from the Centre Hospitalier Universitaire de Sherbrooke (CHUS), thanks to Dr Simon Lévesque (Sherbrooke, QC, Canada). Brain Heart Infusion (BHI), Tryptose–Yeast extract (TY: 3% tryptose and 2% yeast extract, pH 7.4), Luria–Bertani (LB), and Mueller–Hinton (MH) broths were purchased from BD Biosciences (Mississauga, ON, Canada). For solid media, 1% (w/v) agar was added to the above media.

### 2.2. Methods

#### 2.2.1. Surface wettability

The sessile drop method was performed to measure static contact angle on aluminum and copper materials using a goniometer coupled to a camera system (First Ten Angstroms, FTA 200). Drops of distilled water controlled at 1 μL were deposited on the surfaces using a microliter syringe. All steps, from the material placement to wettability measurements using the ellipse–tangent fit method, were automated and controlled by computer *via* FTA32Video software. Four measurements were taken at different locations on both sides of each sample at room temperature allowing consistent assessment of the materials.

#### 2.2.2. Characterization of the anodization layer

A cross section was made on the materials using a cutter with diamond blades from Buehler (Esslingen, Germany). Then, the materials were held vertically in a resin (EpoxyCure™2, Buehler, IL, USA) to be able to observe their cross sections. A polishing (from 1 μm to 0.05 μm) and a metallization step with a gold/palladium mixture were then carried out. Subsequently, the slices of materials were observed by scanning electron microscopy at a voltage of 5.0 kV (Hitachi SU8000, Japan).

#### 2.2.3. QACs release kinetics from anodized aluminum materials with or without impregnation

Material samples (disks of 1 cm in diameter) consisting of AA, AA–AgNO_3_, AA–QACs and AA–AgNO_3_–QACs were immersed in 1 mL of nanopure water for a predetermined duration ranging from 30 s to 144 h. At each time point, the solution was collected, and the absorbance was measured at a wavelength of 215 nm using a UV-VIS spectrophotometer (Ultrospec 2100 pro UV-VIS Spectrophotometer, Amersham Biosciences, UK). Calibration curves obtained by measuring the absorbance at 215 nm of several dilutions of the QACs stock solution showed linear behavior and were used to calculate the concentrations of QACs released from the materials.

#### 2.2.4. Bacteria culture

Frozen stocks of bacterial strains stored at - 80 °C in glycerol were spread out on agar plates and grown overnight under the appropriate conditions (see Table S1†). For *C. difficile* experiments, bacteria were manipulated and incubated under anaerobic conditions (10% hydrogen, 5% CO_2_ and 85% nitrogen) using an anaerobic chamber (Coy Laboratories, Grass Lake, MI, USA), and all media were pre-reduced overnight (O/N) before use. Prior to each experiment, bacterial pre-cultures inoculated from a single isolated colony were prepared in 5 mL of broth and incubated O/N at 37 °C. Then, at their logarithmic stage of growth, bacterial cells were diluted in fresh broth and the optical density at 600 nm (OD_600_) was determined using a portable spectrophotometer (Fisherbrand™ Cell Density Meter 40, ThermoFisher Scientific, USA) to adjust the working bacterial density.

#### 2.2.5. Minimal inhibitory concentration (MIC) and minimal bactericidal concentration (MBC) assay

The minimum inhibitory concentration (MIC) and minimum bactericidal concentration (MBC) of each solution used for the impregnation of materials (AgNO_3_, QACs and AgNO_3_ + QACs solution) were evaluated on each bacterial strain under study. To determine the MIC, doubling dilutions of the solutions to be tested were prepared in 96-well microtiter plates. An inoculum of 100 μL of bacterial suspension (10^6^ colony forming units (CFU) per mL) prepared as mentioned above, was added to each well containing 100 μL of antibacterial solution. Negative controls without bacterial culture and positive controls without antibacterial solution were used in parallel. The plates were incubated for 18 h at 37 °C and the OD_600_ was measured using a UV-VIS spectrophotometer (Synergy™ HTX Multi-Mode Microplate Reader, BioTek, USA). The lowest concentration of each impregnation solution at which no turbidity could be observed was defined as the MIC. To determine the MBC, 100 μL from each well of MIC plates showing no bacteria growth were transferred in 96-well microtiter plates to make decimal dilutions using fresh sterile broth. Subsequently, 20 μL aliquots were spotted on agar plates (spot plating assay^41,42^) and colonies were counted after 24 h of incubation at 37 °C. The lowest concentration of antibacterial solution for which no colony could be observed was defined to be MBC.

#### 2.2.6. Antibacterial activity of materials using a swab liquid-inoculation assay

**Fig. 1:**
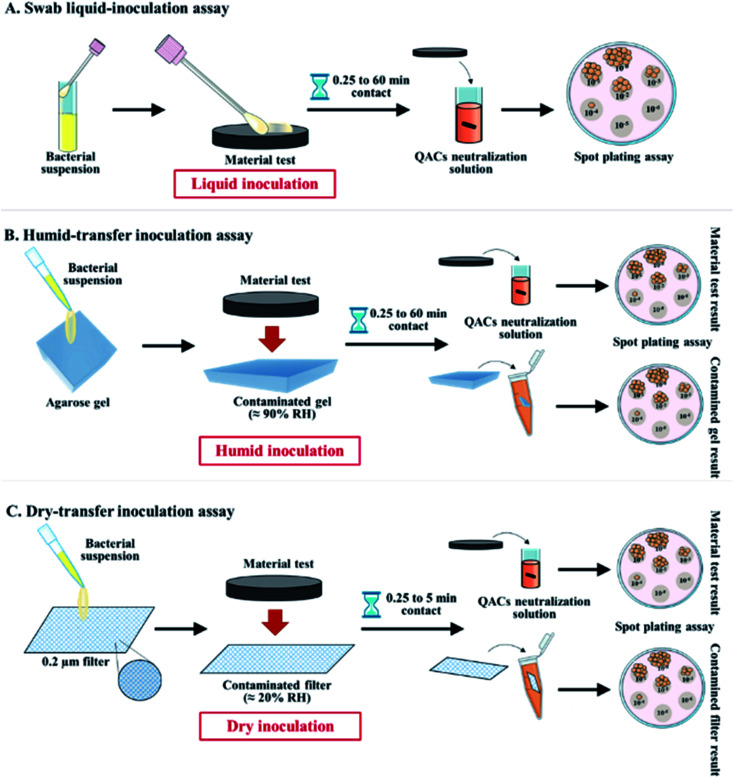
Schematic representation of (A) the swab liquid-inoculation assay, (B) the humid-transfer inoculation assay and (C) the dry-transfer inoculation assay.

A swab liquid-inoculation assay was carried out to evaluate the antibacterial activity of materials (Fig. 1A). A sterile nylon swab (FLOQSwabs™, Copan, Italy) was immersed in a bacterial suspension (10^8^ CFU mL^−1^) prepared as mentioned above and the excess liquid was drained. The contaminated swab was rubbed on the material sample for 5 s, allowing for standardized inoculation of 10^6^ CFU per surface. Following a contact kinetics of 0.25, 1, 5, 15 and 60 min, the contaminated materials were immersed into 1 mL of QACs neutralization solution Casein peptone Lecithin Polysorbate Broth^43,44^ (Sigma-Aldrich®, MO, USA) containing 0.04% of Tween20® (Bioshop®, ON, Canada), and vortexed for 10 s. This neutralization solution did not affect the viability of bacteria. Aliquots of bacteria released from the contaminated materials in the neutralization solution were immediately transferred to a 96-well plate, serially diluted in sterile broth, and spotted on agar plates using a spot plating assay as described above. The number of CFU were counted after O/N incubation at 37 °C to determine the bacterial load present on the materials.

#### 2.2.7. Antibacterial activity of materials using a humid-transfer inoculation assay

A humid-transfer inoculation assay was carried out to evaluate the effectiveness of materials to prevent bacterial cross-contamination under limited water content (Fig. 1B). A 10 μL inoculum of a bacterial suspension (10^8^ CFU mL^−1^) was deposited on a sterile piece of 2% (w/v) agarose gel (1 cm × 1 cm) and left for 1 h under the biological hood to obtain a final relative humidity (RH) of 89.4 ± 0.9%. The RH of the gel was measured by the drying technique in an oven maintained at 160 °C, by means of weighing the gel before and after drying (considered dry when its weight was constant in the oven). To evaluate the level of contamination of the materials resulting from the transfer of bacteria from the gel, the different materials were deposited on the contaminated gels for 0.25, 1, 5, 15 and 60 min and then the number of surviving bacteria on the materials was determined as described above. In parallel, to determine the effect of a contact with antibacterial materials on the bacteria present in the contaminated gel, the pieces of gel were transferred in 2 mL microtubes containing 1 mL of neutralization solution and four sterile 3 mm glass beads. The pieces of gel were then disrupted and homogenized for 10 min at 30 s^−1^ frequency using a Mixer Mill MM 400 (Retsch®, France) to release bacteria trapped in the gel, which were then quantified by spot plating assay as described above. CFUs were counted to determine the number of surviving bacteria remaining in the contaminated gel and after transfer onto the materials.

#### 2.2.8. Antibacterial activity of materials using a dry-transfer inoculation assay

A dry-transfer inoculation assay was performed to evaluate the effectiveness of materials to prevent bacterial cross-contamination under low humidity conditions representative of the surface of the skin (Fig. 1C). Aliquots of 10 μL from a bacterial suspension (10^8^ CFU mL^−1^) were deposited on 0.2 μm sterile nylon filters (Nylon membrane disk diameter 13 mm – filter 0.22 μm, GVS North America, USA). The inoculated filters were dried for 7 min under the biological hood in order to obtain a RH of 16.8 ± 2.6%, representative of the RH found in *Stratum Corneum* of human skin epidermis.^45^ The RH of the filter was measured by the oven drying technique (160 °C) as mentioned above. The different materials were deposited on the inoculated filters for 0.25, 1, 5, 15 and 60 min. Then, the number of surviving bacteria on the contaminated filter and after transfer on the materials were quantified as described above.

#### 2.2.9. Effectiveness of bactericidal materials after washing with cleaning products

The impact of serial washes on the antibacterial properties of the materials copper, Al, AA and AA–AgNO_3_QACs was evaluated. The materials underwent series of 0, 5 or 10 washes using different solutions, including cleaning products commonly used in hospitals: (a) sterile nanopure water, (b) ethanol (70% v/v), (c) Virox™5 containing ≃7% v/v of hydrogen peroxide (utilization: diluted 1/16; v/v), (d) QACs-based impregnation solution (diluted 8 mL in 1 L). When applicable, each wash was performed following the procedure called “disinfection of medical devices” recommended by the cleaning companies. The washes carried out with Virox™5 (The Butcher Company, WI, USA) and the QACs solution were applied to the materials for 5 and 10 min, respectively. The product was dried in air and a water wash was carried out to eliminate the residues of cleaning products on the materials. The washes with sterile nanopure water and ethanol were performed directly, without rinsing. Following these series of washes, the antibacterial efficiency of the materials was evaluated following the swab test methodology, using *S. aureus* and a constant inoculum-material contact time of 1 h. In parallel, copper, Al, AA and AA–AgNO_3_–QACs without wash with cleaning solution were used as controls.

#### 2.2.10. Efficiency of A3S materials following immersions in water

AA–AgNO_3_–QACs underwent series of 1 to 10 successive 12 h immersions in 1 mL of sterile nanopure water. Between each successive immersion, the used deionized water was replaced with 1 mL of fresh sterile nanopure water. Following these series of immersions, the antibacterial efficiency of the AA–AgNO_3_–QACs was evaluated with the swab liquid-inoculation methodology, using *S. aureus* and a constant inoculum-material contact time of 1 h. In parallel, the same experiment was repeated using AA control samples and AA–AgNO_3_–QACs treated samples which had not been immersed in sterile nanopure water.

#### 2.2.11. Statistical analyses

Analysis of variance (ANOVA) and subsequent statistical tests (Tukey–Kramer studentized range post-hoc tests) were performed using Excel (Excel 2013®) and GraphPad (GraphPad Software 2020 Inc., Prism 8, San Diego, CA, USA). Only difference with a *p* < 0.05 were considered significant. In the current paper, the number of independent experiments is defined as “*n*” and the total number of technical replicates per condition is defined as “*N*”.

## 3. Results and discussion

### 3.1. Characterization of anodized aluminum materials

All the anodized aluminum-based materials (with or without impregnation) used in this study were prepared and provided by the company A3S (Fig S1†).

#### 3.1.1. Surface characterization

##### Hydrophobicity by contact angle measurement

Since wettability of surfaces can influence the first stage of pathogen adhesion on materials,^46,47^ static contact angle measurements were carried out using a goniometer to assess the surface wettability of Al, AA, AA–AgNO_3_, AA–QACs, AA–AgNO_3_–QACs and copper. As shown in Table 1, the reference materials copper and Al exhibited a slight hydrophobicity of their surface (around 78–85°), in accordance with previously published data.^48–51^

**Table 1:** Surface wettability of the materials using contact angle measurement by goniometer

Material	Contact angle measurement in static condition^a^ (°)	Significant effect^b^ compared to	
		Al	AA
Copper	78.05 ± 3.77	—	**
Al	85.53 ± 2.83	—	**
AA	48.99 ± 6.76	**	—
AA–AgNO_3_	45.96 ± 5.53	**	—
AA–QACs	67.25 ± 4.00	*	*
AA–AgNO_3_–QACs	69.37 ± 4.49	*	*

a Results are means ± SD (*n* = 2; *N* = 24).

b Significant effect *p < 0.01 and **p < 0.001.

In contrast, the anodization process on Al significantly decreased the contact angle (around 49°), the AA being more hydrophilic than Al (*p* < 0.001). These results are in contradiction with the hydrophobic/superhydrophobic properties normally induced by the hard anodization process of aluminum materials.^52–54^

However, this difference was probably induced during the final stage of manufacturing AA materials by the company A3S, which consists of pore sealing process by hydration of alumina molecules on the surfaces leading to dilatation and gradual closure of the alumina oxide layer.^55^ Furthermore, the impregnation step with QACs modified the wettability of AA. Indeed, unlike AA–AgNO_3_, which showed similar contact angle in comparison with non-impregnated AA, AA–QACs partially restored the hydrophobicity lost following anodization (*p* < 0.001). QACs cations are composed of a positively charged nitrogen atom with four long non-polar carbon chains, which, used as a coating, can influence the surface hydrophobicity of the material.^56–59^ Thus, the type of solution used during the material impregnation step influences their surface wettability property. Generally, super-hydrophobic materials (*θ* > 150°) are recommended for antibacterial applications, since they inhibit cellular adhesion.^60^

##### Observation of the anodization layer by scanning electron microscopy

**Fig. 2:**
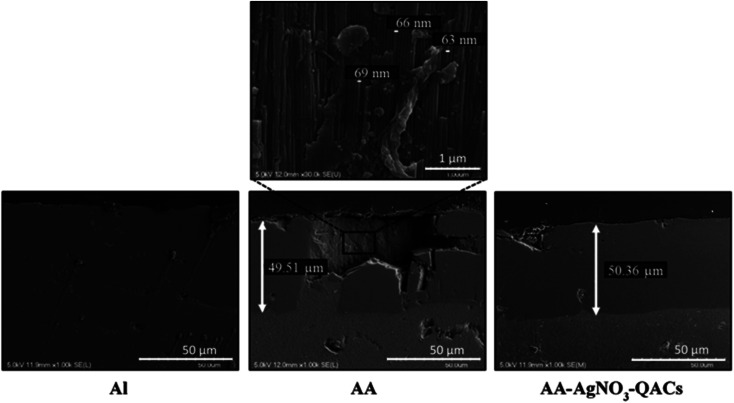
Characterization of the anodized layer of reference materials using cross sectional observation by scanning electron microscopy. Results are representative of two independent experiments and four replicates.

Cross sections were carried out to characterize by scanning electron microscopy the anodization layer of AA with or without AgNO_3_–QACs impregnation (Fig. 2). As expected, untreated aluminum Al did not show any layer at its surface. However, hard anodized AA and AA–AgNO_3_–QACs possessed over all of their surface a homogeneous layer of anodization with a thickness of about 50 μm. This layer was composed of organized arrays of pores with an overall diameter of 65–70 nm (the narrowing of the diameter of pores on anodized aluminum materials, due to the clogging stage, is not considered in these analyses) (Fig. 2). These results agree well with those of other studies showing the formation of an anodized layer thickness ranging from 25 to 100 μm with pore diameters between 58 and 134 nm depending on the operating parameters (acid solution, temperature, current density).^53,61,62^

Hard anodization, consisting in electrolysis in a sulfuric acid solution (H_2_SO_4_: 1–15% w/v), is performed at a low temperature (between −5 and 5 °C) with a high current density (20 mA cm^−2^) and an anodization time varying between 60 to 90 min.^63,64^ Therefore, bacillus-shaped (≃1.0 μm wide by 3.0 μm long) and coccus-shaped (≃ 1.0 μm diameter) bacteria would be in contact with ≃780 and 200 pores, respectively, when deposited on these surfaces.

#### 3.1.2. Release kinetics of antibacterial agents impregnated on anodized aluminum materials

##### Quaternary ammonium compounds (QACs) release kinetics

**Fig. 3:**
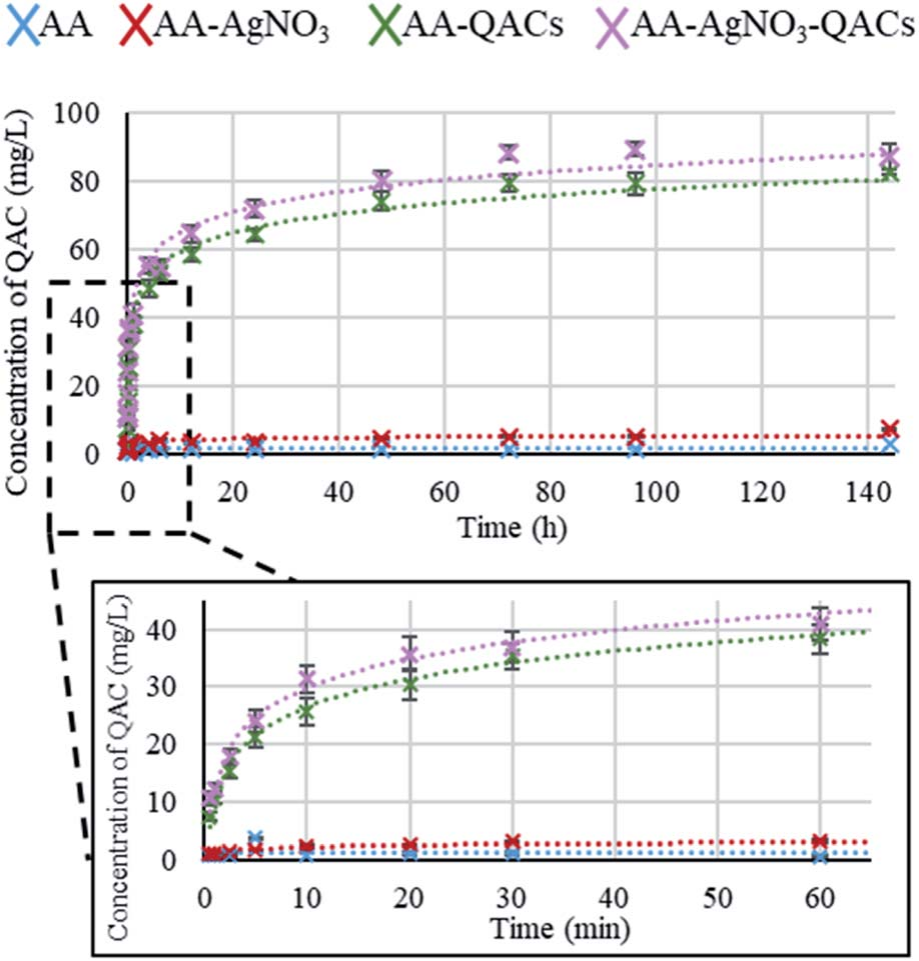
Amount of QACs released (mg L^−1^) from anodized aluminum materials after immersion in nanopure water for different periods of time. Results are means ± SD (*n* = 4; *N* = 12).

The release kinetics of the QACs were carried out on AA, AA–AgNO_3_, AA–QACs and AA–AgNO_3_–QACs (Fig. 3). The quantification of QACs release was calculated from a standard curve (Fig. S2†). As expected, no release of QACs was detected from both AA (negative control) and AA–AgNO_3_. The release kinetics for AA–QACs and AA–AgNO_3_–QACs were very similar and showed an extremely fast QACs release, from the first minutes of immersion in water, followed by a slower release up to 72 h of immersion (around 20 and 40 mg L^−1^ of QACs released after 5 min and 1 h of immersion, respectively). These two distinct phases are characteristic of release patterns from nanoporous anodized aluminum structures in non-agitated systems.^65,66^

Therefore, the use of these materials in liquid environments (immersed) is not recommended.

##### Silver ion (Ag^+^) release kinetics

Tests using ICP-OES, allowing an ion detection level of 23 ppb, were carried out to assess the release of Ag^+^ from AA–AgNO_3_–QACs. No Ag^+^ could be detected following an immersion for up to 1 h and 24 h, which is consistent with the study carried out by Valiei *et al.*^36^ Hence, the impact of Ag^+^ on the antibacterial activity of the impregnated surfaces is expected to be minimal compared to that of QACs, as suggested also by our Minimal Inhibitory Concentration (MIC) and Minimal Bactericidal Concentration (MBC) assays.

### 3.2. Minimal Inhibitory Concentration (MIC) and Minimal Bactericidal Concentration (MBC) assay

Before assessing the antibacterial efficiency of impregnated materials, it was essential to first characterize the activity of antibacterial agents selected for impregnation of the surface materials. The MIC and MBC of (i) AgNO_3_ (1% w/v), (ii) QACs-based (10.9% w/v) solution and (iii) a combination of both AgNO_3_ (1% w/v) and QACs-based (10.9% w/v) solutions were determined on the pathogenic bacteria *S. aureus*, *C. difficile*, vancomycin-resistant *E. faecium* and *E. faecalis*, *E. coli*, streptomycin-resistant *S. typhimurium* and *K. pneumoniae* after 18–24 h incubation (Table 2).

**Table 2:** MIC and MBC of impregnation solutions (AgNO_3_, QACs and AgNO_3_ + QACs) on different Gram-positive and Gram-negative bacteria stains^a^

		AgNO_3_ (mg L^−1^ of AgNO_3_)		QACs (mg L^−1^ of QACs)		AgNO_3_ + QACs (mg L^−1^ of QACs)	
		MIC	MBC	MIC	MBC	MIC	MBC
Gram-positive	*S. aureus*	35.40 ± 5.91	95.10 ± 15.80	0.22 ± 0.03	1.62 ± 0.29	0.27 ± 0.03	1.88 ± 0.23
	*C. difficile*	58.60 ± 5.03	87.83 ± 10.96	0.87 ± 0.09	1.19 ± 0.17	0.94 ± 0.10	1.11 ± 0.08
	Vancomycin-resistant *E. faecium*	28.09 ± 3.33	63.43 ± 13.95	0.98 ± 0.09	1.53 ± 0.19	0.98 ± 0.09	1.53 ± 0.19
	Vancomycin-resistant *E. faecalis*	90.22 ± 17.19	302.90 ± 55.42	1.02 ± 0.09	1.02 ± 0.09	1.06 ± 0.09	1.36 ± 0.15
Gram-negative	*E. coli*	5.65 ± 0.88	11.59 ± 1.66	2.56 ± 0.25	3.07 ± 0.38	3.92 ± 0.35	4.09 ± 0.35
	Streptomycin-resistant *S. typhimurium*	25.63 ± 2.35	42.73 ± 5.70	3.03 ± 0.40	4.94 ± 1.19	3.75 ± 0.34	7.84 ± 1.76
	*K. pneumoniae*	13.42 ± 1.22	25.63 ± 2.81	3.41 ± 0.30	3.41 ± 0.30	3.41 ± 0.30	3.41 ± 0.30

a Results are means ± SEM (*n* = 4; *N* = 16).

The mechanism of action of AgNO_3_ is induced by the release of Ag^+^ in solution. These ions bind to the thiol groups of proteins in cell membranes, enzymes and DNA, causing their denaturation and affecting their functions.^67,68^ For all the bacteria, MIC and MBC obtained with the AgNO_3_ solution varied between 5.65 and 90.22 mg L^−1^ and between 11.59 and 302.90 mg L^−1^, respectively (Table 2). These values are in accordance with the MIC range identified in the literature for *S. aureus*, *E. coli* and vancomycin-resistant enterococci.^69–72^ MIC and MBC values were also significantly higher for Gram-positive bacteria (MIC between 28.09 and 90.22 mg L^−1^ and MBC between 63.43 and 302.90 mg L^−1^) than for Gram-negative bacteria (MIC between 5.65 and 25.63 mg L^−1^ and MBC between 11.59 and 42.73 mg L^−1^) (*p* < 0.0001; Table 2). On the other hand, the MIC and MBC values for bacteria resistant to vancomycin (*E. faecium* and *E. faecalis*) or streptomycin (*S. typhimurium*), were similar to other bacteria of their Gram-positive and Gram-negative groups, respectively (Table 2).

QACs mainly damage the cytoplasmic membrane of bacteria by disrupting the lipid bilayers through the alkyl chains of QACs molecules.^73^ This mode of action is attributed to various factors such as molecular weight, molecular charge density or the length of the QACs *N*-alkyl chains.^74–82^ The evaluation of the QACs solutions showed MIC ranging from 0.22 to 3.41 mg L^−1^ and MBC ranging from 1.02 to 4.94 mg L^−1^, coinciding perfectly with values found in the literature for chlorinated QACs (0.25 to 5.0 mg L^−1^).^83–86^ A significant difference between Gram-positive and Gram-negative bacteria was observed for the QACs solution and was inversed with what was observed for the AgNO_3_ solution. Indeed, MIC and MBC values were significantly lower for Gram-positive bacteria (MIC between 0.22 and 1.02 mg L^−1^ and MBC between 1.02 and 1.62 mg L^−1^) than for Gram-negative bacteria (MIC between 2.56 and 3.41 mg L^−1^ and MBC between 3.07 to 4.94 mg L^−1^) (*p* < 0.0001; Table 2).

Although QACs are broad-spectrum antibacterial agents against Gram-positive and Gram-negative bacteria, the cell wall of Gram-positive cells is composed of a single plasma membrane, generally making them more sensitive to QACs than Gram-negative bacteria that possess two lipid bilayers.^87,88^

In addition, for all the tested bacteria, MIC and MBC obtained with the AgNO_3_ solution were significantly higher (100 to 1500 times) than those obtained with the QACs solutions (*p* < 0.0001), demonstrating the strong antibacterial efficiency of QACs on Gram-positive and Gram-negative bacteria. This likely explains why MIC and MBC values with the AgNO_3_ + QACs combination were quite similar to those obtained with the QACs alone (Table 2).

All the results described above were performed on vegetative bacteria, *i.e.*, metabolically active bacteria. Since bacterial spores represent important vehicles for dissemination of certain bacterial pathogens, the sporicidal potential of the impregnating solutions was assessed on *C. difficile* spores (≃1.6 × 10^5^ spores per mL), following contact times of 1, 4 and 24 h. The sporicidal disinfectant Virox™5 was used as a comparison control (Fig. S3†). The results showed that none of the QACs-containing solutions (QACs alone or AgNO_3_ + QACs) induced a significant decrease in the number of *C. difficile* spores, as opposed to the sporicidal controls (1/16 (v/v): Virox™5/water) for which no viable spores were detected (Fig. S3†). These results are in agreement with the literature showing that QACs are generally non-sporicidal.^89^

However, in this study, all the vegetative bacteria showed great sensitivity to the QACs-containing solutions, thus justifying the interest of their use for manufacturing antibacterial surfaces.

### 3.3. Assessing the antibacterial properties of materials on different pathogenic bacteria

**Fig. 4:**
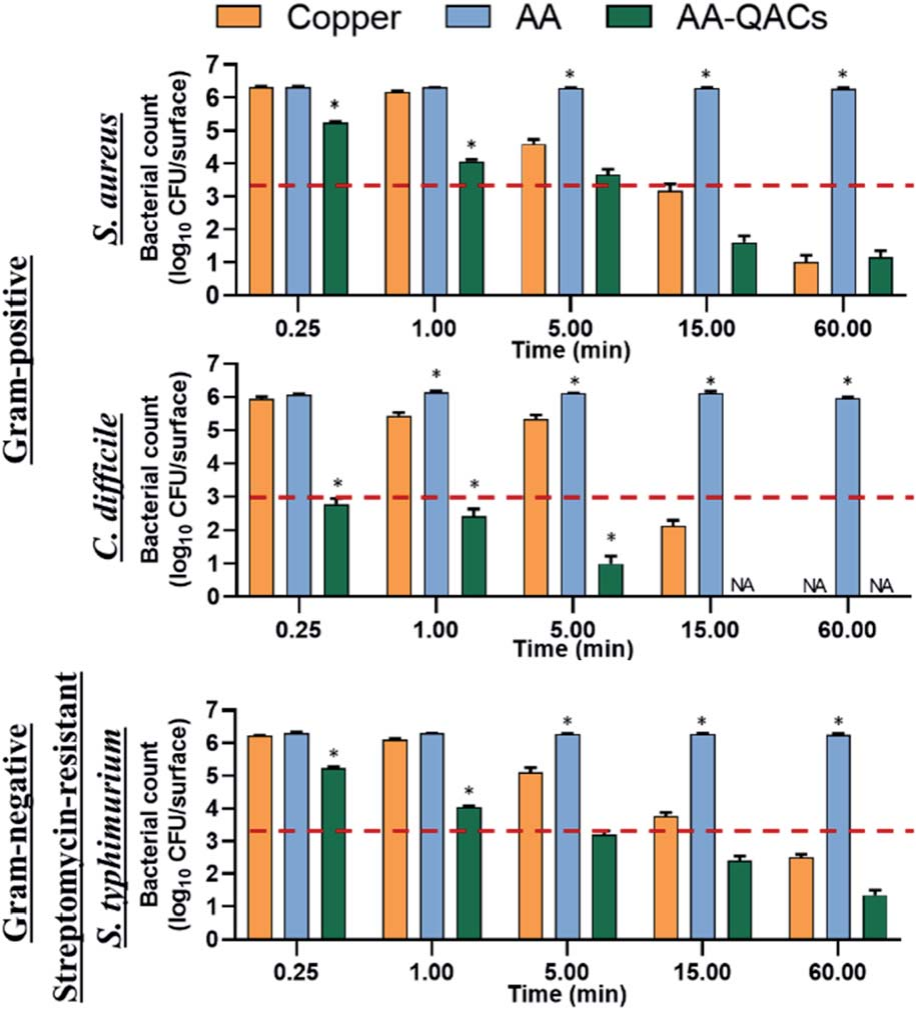
Swab liquid-inoculation assay evaluating the antibacterial activity of different materials. Results are means ± SEM (*n* = 2; *N* = 8). The reference of 99.9% antibacterial activity is indicated by the dotted red line. Statistical effect compared to copper: *p < 0.001. NA: no bacteria counted.

**Fig. 5:**
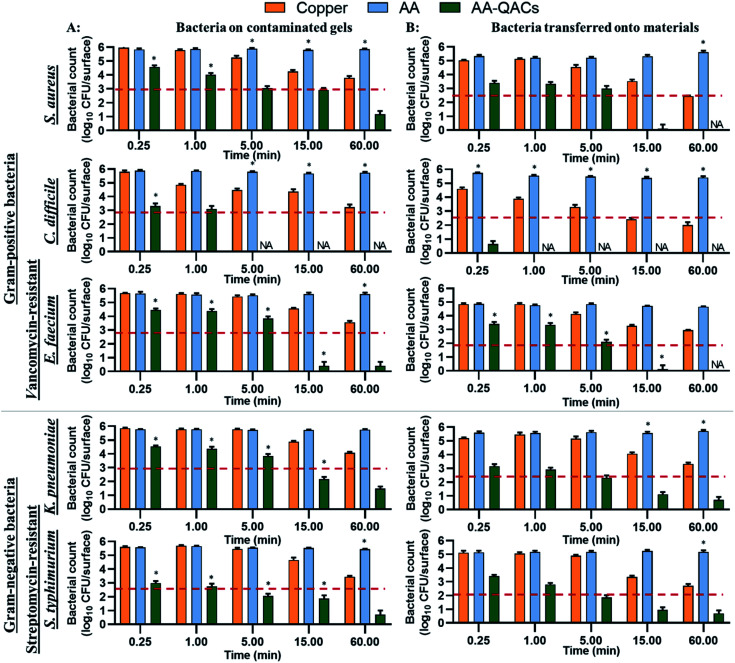
Humid-transfer inoculation assay from contaminated gel evaluating the antibacterial activity of materials. The graph shows total counts of bacteria that survived (A) on the contaminated gel or (B) after transfer from the gel to the different materials. The reference of 99.9% antibacterial activity is indicated by the dotted red line. Results are means ± SEM (*n* = 2; *N* = 8). Statistical significance when compared to copper: *p < 0.001. NA: no bacteria counted.

**Fig. 6:**
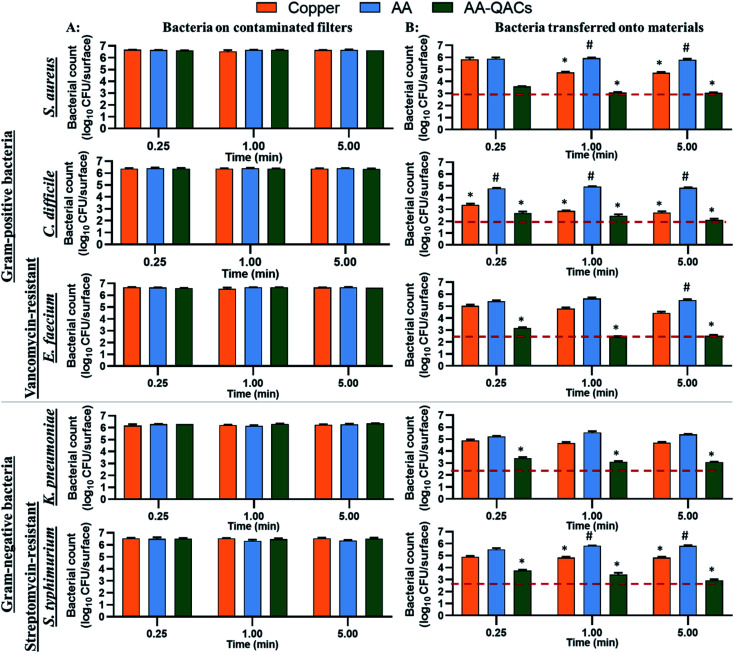
Dry-transfer inoculation assay evaluating the antibacterial activity of materials after contact with a contaminated filter under low humidity conditions. Bacterial counts in log_10_ CFU per surface are indicated for (A) contaminated filters and (B) after transfer onto materials. Results are means ± SEM (*n* = 2; *N* = 8). The reference of 99.9% antibacterial activity is indicated by the dotted red line. Statistical effect compared to AA: *p < 0.001 and to copper: #*p* < 0.001.

The antibacterial activity of materials was evaluated against both Gram-positive (*S. aureus*, *C. difficile*, vancomycin-resistant *E. faecium*) and Gram-negative pathogens (streptomycin-resistant *S. typhimurium* and *K. pneumoniae* possessing a capsule) following contact times from 0.25 to 60 min. In addition, we compared three different inoculation methods (Fig. 1): (i) a swab liquid-inoculation assay (Fig. 4), (ii) a humid-transfer inoculation assay from a contaminated gel (Fig. 5) and (iii) a dry-transfer inoculation assay from a contaminated filter (Fig. 6). These different methodologies were chosen to allow comparison of the antibacterial activity of materials under different humidity conditions, to mimic wet (fomites, aerosols) and dry (contaminated hands of patients, medical equipment or other inert objects) environments associated with transmission of bacterial pathogens.^90–92^Fig. 4–6 show the results with copper (antibacterial control), AA (negative control) and AA–QACs, while results obtained with Al, AA–AgNO_3_ (identical results to the AA control) and AA–AgNO_3_–QACs (identical results to AA–QACs) are presented in ESI (Fig. S4–S6†).

#### 3.3.1. Antibacterial activity of materials using swab liquid-inoculation assay

The initial number of bacteria recovered from negative control AA was ≃1.6 × 10^6^ CFU per surface, as determined after a 0 min contact time. This inoculum was sufficient to observe a 3-log decrease in bacterial viability (99.9% antibacterial activity).^43^ Bacterial counts were stable on both Al and AA negative control materials with ≃1.6 × 10^6^ CFU per surface within 60 min for all tested bacteria (Fig. 4 and S4†), showing that the anodization process alone did not confer antibacterial property on Al. Compared to AA, copper showed a significant decrease in bacterial counts (99.9%, corresponding to a 3-log CFU per surface decrease) after 15 min with *S. aureus* and *C. difficile* (1.5 × 10^3^ and 1.4 × 10^2^ CFU per surface, respectively), and after 60 min with *S. typhimurium* (3.1 × 10^2^ CFU per surface) (Fig. 4). These values are in agreement with the well-known antibacterial properties of copper (C70600 alloy), inducing 99.9% antibacterial activity after 15 and 30 min of contact with *E. coli* and *S. aureus*, respectively.^93,94^ Another study showed that against *C. difficile*, copper showed the same antibacterial activity (99.9%) after 240 min.^95^

AA–AgNO_3_ had no significant effect on bacterial viability for all contact times, demonstrating that impregnation with an AgNO_3_ (1% w/v) solution did not induce antibacterial activity on AA within 60 min (Fig. S4†). This was expected based on our MIC and MBC assays (Table 2), as well as the release kinetics assays (Fig. 3). In contrast, the AA–QACs caused a greater bacterial decrease than AA and copper after 0.25 and 1 min of contact, for all the bacteria (*p* < 0.001: Fig. 4). For example, AA–QACs showed 99.9% antibacterial activity after 0.25 min for *C. difficile* (*p* < 0.001: 6.0 × 10^3^ CFU per surface), 5 min for *S. typhimurium* (*p* < 0.001: 1.5 × 10^3^ CFU per surface) and 15 min of contact with *S. aureus* (*p* < 0.001: 4.1 × 10^1^ CFU per surface). The antibacterial effect of AA–QACs was even more pronounced after longer contact times, decreasing the quantity of surviving bacteria to <2.0 × 10^1^ CFU per surface after 5 min for *C. difficile* (1.04 × 10^1^ CFU per surface) and 60 min for *S. aureus* and *S. typhimurium* (1.4 × 10^1^ and 1.9 × 10^1^ CFU per surface, respectively) (Fig. 4). Finally, similar results to AA–QACs were obtained with AA–AgNO_3_–QACs (Fig. S4†).

Although we did not observe sporicidal activity in MIC and MBC assays with the various impregnation solutions (Fig. S3†), we also tested the sporicidal activity of the different materials on *C. difficile* spores after 1 and 24 h of contact. As expected, no sporicidal effect could be observed when compared to the AA negative control (Fig. S4†).

In summary, under liquid conditions using the swab test, anodized aluminum materials impregnated with QACs showed a faster and stronger antibacterial activity against both Gram-positive and Gram-negative bacteria compared to copper, whose alloy is approved as an antibacterial reference by EPA.^39^ In addition, the impregnation with AgNO_3_, alone or in combination with QACs, did not improve the overall activity of materials (Fig. S4†). Therefore, consistent with the MIC and MBC results, the effect of AgNO_3_ compared to QACs appeared to be negligible in this study.

#### 3.3.2. Antibacterial activity of materials using a humid-transfer inoculation assay

We next wanted to assess the antibacterial properties of the different materials using a humid-transfer protocol, to mimic a wet contamination (fecal spills, contaminated aerosols, or food). To do this, we developed a gel contamination assay, which recreates conditions of high humidity, but without excessive liquid like with the swab method. Contact kinetics of 0.25, 1, 5, 15 and 60 min were done with *S. aureus*, *C. difficile*, vancomycin-resistant *E. faecium*, streptomycin-resistant *S. typhimurium* and *K. pneumoniae* (Fig. 5). The number of bacteria at time zero on contaminated gels was ≃6.0 × 10^5^ CFU cm^−2^, allowing a sufficient bacterial inoculum deposit on the materials to assess their antibacterial properties (6.0 × 10^4^ and 4.0 × 10^5^ CFU per surface depending on the type of bacteria).^43^ The number of bacteria was stable over time on both AA and Al negative controls and their contaminated gels (Fig. 5 and S5†).

The copper induced a significant decrease of 99% in bacterial counts (2-log CFU cm^−2^ decrease) on contaminated gels compared to AA after 60 min of contact with *S. aureus*, *C. difficile*, *E. faecium* and *S. typhimurium* (*p* < 0.001, Fig. 5A). In contrast, copper had little antibacterial activity on gels contaminated with *K. pneumoniae* (not significant). The low efficiency of copper on *K. pneumoniae* may be explained by the fact that this bacterium produces a thick polysaccharide capsule which provides protection against antibacterial agents, in addition to contributing to its virulence.^96–98^ However, when assessing bacterial survival after transfer from the gel to the surface of the different materials, copper induced a significant decrease of 99.9% in *S. aureus* and *C. difficile* counts (*p* < 0.001) and of 99% in counts of *K. pneumoniae*, *S. typhimurium* (*p* < 0.001) and *E. faecium* (*p* < 0.01) after a contact time of 60 min (Fig. 5B). The release of copper ions is considered to be the key mechanism of antibacterial activity of these materials.^99,100^ In the humid-transfer inoculation assay, the reduced water content limits the release of copper ion, which might explain why copper presented a lower activity with the gel inoculation protocol than with the swab or international standards methodology.^39,43^

Furthermore, while AA and AA–AgNO_3_ gave results similar to Al (Fig. S5†), AA–QACs had a greater antibacterial activity on contaminated gels after 0.25 min of contact for all bacteria, compared to the copper control (*p* < 0.001: Fig. 5A). For example, the AA–QACs showed 99.9% antibacterial activity on contaminated gels after 5 min for *C. difficile* and *S. typhimurium* (*p* < 0.001: 0 and 1.1 × 10^2^ CFU per surface, respectively), and 15 min for *S. aureus*, *E. faecium* and *K. pneumoniae* (*p* < 0.001: 8.2 × 10^2^, 2.5 × 10^0^ and 1.5 × 10^2^ CFU per surface, respectively). The antibacterial activity of AA–QACs was greater and faster following transfer of bacteria onto materials (<1.0 × 10^2^ CFU per surface; Fig. 5B), resulting in 99.995% of bacteria killed after 0.25 min of contact with *C. difficile* (*p* < 0.001: 4.2 × 10^1^ CFU per surface) and after 15 min of contact with *S. aureus*, *E. faecium*, *K. pneumoniae* and *S. typhimurium* (*p* < 0.001: 1.2 × 10^0^, 1.3 × 10^0^, 1.3 × 10^1^ and 8.8 × 10^1^ CFU per surface, respectively).

Finally, AA–AgNO_3_–QACs showed a great efficiency, equivalent to AA–QACs, confirming that the addition of AgNO_3_ did not improve the overall activity of the materials (Fig. S5†).

#### 3.3.3. Antibacterial activity of materials using a dry-transfer inoculation assay

The dry contamination of high-touch surfaces, for example through contaminated hands, tissues, medical devices or other objects, actively participates in the transmission of pathogenic bacteria in both hospital and community environments.^90–92^ Therefore, tests mimicking bacterial dry-transfer were carried out to assess the killing efficiency of antibacterial materials in a similar context. To this end, we developed a filter contamination assay that recreates low humidity conditions (<20% RH). Contact times of 0.25, 1 and 5 min were used in assays with *S. aureus*, *C. difficile*, vancomycin-resistant *E. faecium*, streptomycin-resistant *S. typhimurium* and *K. pneumoniae* (Fig. 6). The number of bacteria at time zero on the contaminated filters was ≃1.0 × 10^6^ CFU cm^−2^. The initial number of bacteria deposited on each material varied between 6.9 × 10^4^ and 7.8 × 10^5^ CFU per surface depending on the bacterial species tested. The initial quantity of inoculum on all contaminated filters, was stable over time (0.25, 1 and 5 min) for all bacteria (Fig. 6A). The different materials tested had no impact on the viability of bacteria present on the filters and the number of bacteria transferred to both AA and Al negative controls remained stable over time for all bacteria species (Fig. 6B). The copper showed only an antibacterial activity of 99% (2-log CFU per surface decrease) after 1 and 5 min of contact with *C. difficile* (*p* < 0.001: 7.4 × 10^2^ CFU per surface) and killed less than 90% (≤1-log CFU per surface decrease) of *S. aureus*, *E. faecium*, *K. pneumoniae* and *S. typhimurium* (Fig. 6). These results differ from those obtained with the gel inoculation protocol, where copper had antibacterial activity greater than 99.9% (≥3 log of CFU per surface decrease) after 60 min of contact (Fig. 5B). This difference can be explained by the fact that contact times ≤ 5 min in dry conditions would not allow the sufficient release of copper ions to induce bacterial death.^99,100^ This demonstrates the relevance of assessing different methodologies and contamination protocols mimicking real conditions to which materials will be exposed to (short contact time, low humidity, *etc.*).^101,102^ Moreover, the number of bacteria transferred to AA–AgNO_3_ was not affected over time for all bacteria (Fig. S6B†). In contrast, AA–QACs induced a significant decrease of more than 99.5% bacteria after 1 min of contact with *S. aureus*, *C. difficile*, *E. faecium*, *K. pneumoniae* and *S. typhimurium* (*p* < 0.001: 1.2 × 10^3^, 2.9 × 10^2^, 2.6 × 10^2^, 1.3 × 10^3^ and 2.5 × 10^3^ CFU per surface, respectively) (Fig. 6). Similar results were obtained with AA–AgNO_3_–QACs (Fig. S6B†).

To summarize, by mimicking liquid and humid transmission routes (swab and humid-transfer assay), responsible for frequent hospital and community contaminations (fecal spills, aerosols, contaminated food), AA containing QACs showed a high and quick antibacterial efficiency (more than 99.95% in less than 15 min) on all pathogenic vegetative bacteria tested, thus exceeding the antibacterial activity required by standardization agencies^43,103^ (Fig. 4 and 5). In addition, by mimicking contamination under dry conditions, these materials showed promising results even for short contact times (*e.g.*, 1 min) and were more efficient than the copper reference (Fig. 6). This high antibacterial performance in short contact times is a highly desired feature in the manufacturing of high-touch antibacterial surfaces since it is crucial to reduce pathogenic transmission linked to the contaminated inert environment. In fact, in public areas with high contact surfaces, such as in hospitals, the average elapsed time between two people touching the same surface is estimated at 5 min.^101,102^ Following humid contact, the AA–QACs allowed the contaminated gels to be “partially sterilized” (99.9% decrease in bacteria in less than 5 or 15 min of contact) (Fig. 5). However, AA–QACs had no impact on the viability of bacteria on the contaminated filters. Thus, the mode of action of AA–QACs appears to require the presence of humidity to induce sufficient release of the active agents from the material, consistent with the rapid release kinetic of QACs observed following material immersion (Fig. 3).

In our assays, the filter was the source of the contamination and it's relative humidity was ≃20%, which is similar to the superficial layers of *Stratum Corneum* in the human epithelium.^45,104^ Under these conditions, the release and transfer of QACs to the filter was negligeable. However, it was sufficient to allow effective and rapid antibacterial activity against bacteria transferred onto the materials. These results suggest that under RH conditions similar to those of the skin, AA–QACs may not have harmful impacts on the human skin or its microbiome (good bacteria colonizing the human skin playing an essential protective role^105^), while enable self-disinfection of the contaminated surfaces. This would constitute a very important advantage since the disruption (dysbiosis) of this microbial balance can affect immune defenses, cause excessive inflammation of the skin tissue and lead to the development of pathologies such as acne or psoriasis.^106,107^ However, the impact of AA–QACs on the microbiome or human skin tissue must be determined in a future study.

### 3.4. Effectiveness of antibacterial materials after washing with cleaning products

**Fig. 7:**
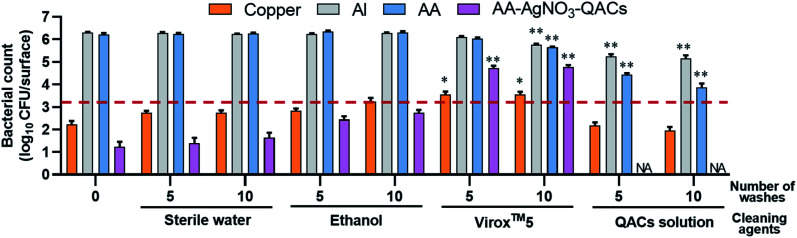
Impact of 0, 5 or 10 consecutive washes with different cleaning solutions on the antibacterial properties of materials tested against *S. aureus*. The reference of 99.9% antibacterial activity is indicated by the dotted red line. Results are means ± SEM (*n* = 3; *N* = 12). Statistical effect compared to the corresponding material without cleaning agent: *p < 0.01 and **p < 0.0001. NA: no bacteria counted.

The inert surfaces in public environments (door knobs, stair railings, support ramps, *etc.*) are regularly cleaned using specific sanitizing and disinfecting agents.^108^ To assess longevity of the AA–QACs materials, the effects of several cleaning products on the antibacterial properties of the materials were evaluated using *S. aureus* and a constant contact time of 1 h (Fig. 7). The tests assessed the impact of 0, 5 and 10 consecutive washes with different cleaning solutions containing: (a) sterile water, (b) ethanol, (c) Virox™5, and (d) a QACs-based commercial solution. No significant decrease in the number of surviving bacteria was observed on the Al and AA controls following 5 or 10 washes with sterile water or ethanol. However, a slight decrease in CFU per surface (1 log reduction), was observed on the Al and AA following 10 washes with Virox™5 (5.8 × 10^5^ and 4.3 × 10^5^ CFU per surface, respectively; *p* < 0.0001) and 5 or 10 washes with the QACs solution (1.7 × 10^5^ and 1.4 × 10^5^ CFU per surface for Al and 2.7 × 10^4^ and 8.7 × 10^3^ CFU per surface for AA, respectively; *p* < 0.0001) (Fig. 7). Antibacterial residues from the series of washes could explain this slight drop in bacterial counts on these negative control materials. Indeed, after their use, the elimination of QACs (cationic surfactant) requires the use of abundant rinsing.^109^ In addition, QACs such as ADBAC and DDAC have extremely low vapor pressures (3.53 × 10^−12^ mm Hg (4.7 × 10^−10^ Pa) and 2.33 × 10^−11^ mm Hg (3.1 × 10^−9^ Pa), respectively).^110,111^ Therefore, the potential residues of these QACs do not volatilize spontaneously and persist on materials.

The antibacterial activity of copper remained relatively unaffected after washes with sterile water, ethanol or QACs solution. Similarly, the antibacterial activity of AA–AgNO_3_–QACs was relatively unaffected by 5 or 10 washes with sterile water or ethanol. However, Virox™5 washes resulted in a significant decrease in the antibacterial activity of copper (3.6 × 10^3^ CFU per surface, *p* < 0.01) and AA–AgNO_3_–QACs (5.4 × 10^4^ CFU per surface, *p* < 0.0001) (Fig. 7). Hydrogen peroxide (H_2_O_2_) is a strong oxidizing agent (*E*_o_ = 1.763 V at pH 0, *E*_o_ = 0.878 V at pH 14).^112^ Thus, it alters the copper by electrochemical dissolution, resulting in a maximum elimination rate of copper with 1% H_2_O_2_ (Virox™5 use concentration ≃0.5% H_2_O_2_).^113^ Moreover, clogging of anodized aluminum materials gives them stability and protection against corrosion and abrasion.^55,114,115^ However, in this study, the AA surface is covered by a nanoporous layer. Hence, H_2_O_2_ can penetrate into the anodized layer, affecting its integrity.

Of note, the washes carried out with the QACs solution improved the antibacterial properties of AA–AgNO_3_–QACs, with no viable counts detectable after 5 washes (Fig. 7). As in previous experiments, results similar to those obtained with AA–AgNO_3_–QACs are expected from AA–QACs materials. In summary, the integrity of the AA impregnated with QACs, and their antibacterial properties must be maintained by using appropriate cleaning solutions (*e.g.*, QACs solution), if repeated washing procedures are to be applied. The added advantage of such cleaning step is that it seems to improve the overall antibacterial efficiency of the materials.

### 3.5. Effectiveness of antibacterial materials following immersions in water

**Fig. 8:**
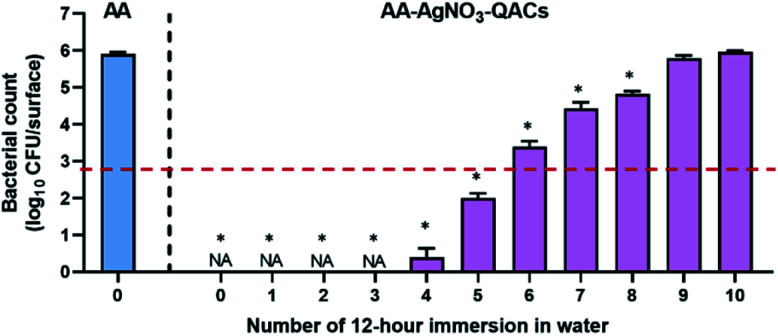
Impact of successive 12 h immersions in sterile water on the antibacterial properties of materials on *S. aureus*. The reference of 99.9% antibacterial activity is indicated by the dotted red line. Results are means ± SEM (*n* = 3; *N* = 12). Statistical effect compared to AA: *p < 0.0001. NA: no bacteria counted.

As demonstrated by our release kinetics assays, immersion of AA–QACs (or AA–AgNO_3_–QACs) in water leads to a rapid release of QACs from the impregnated surfaces. To assess the impact of water immersion on the antibacterial activity of AA–AgNO_3_–QACs, and to determine the suitable applications for these materials, we tested the impact of a series of successive 12 h immersions in water. The swab liquid-inoculation assay was performed on *S. aureus* with a constant contact time of 1 h (Fig. 8). Without immersion, the AA–AgNO_3_–QACs showed a significant 99.9999% antibacterial activity compared to AA (*p* < 0.001). No surviving bacteria could be detected on these materials after 1 h of contact. Similar results were observed after 1, 2, and 3 rounds of immersions (≃0 CFU per surface, *p* < 0.001). Following 4 and 5 rounds of immersion, the antibacterial activity of the AA–AgNO_3_–QACs decreased slightly (1.0 × 10^2^ CFU per surface after 5 rounds), while still maintaining a great efficiency by eliminating 99.99% of bacteria. However, after 6, 7, and 8 rounds of immersion, the AA–AgNO_3_–QACs surfaces gradually lost their antibacterial activity (2.4 × 10^3^, 2.7 × 10^4^, 6.7 × 10^4^ CFU per surface, respectively) (Fig. 8). After 9 and 10 immersions, the materials completely lost their antibacterial properties and were comparable to the AA control (6.2 × 10^5^ and 9.0 × 10^5^ CFU per surface; Fig. 8). This phenomenon is likely explained by the fact that following immersion in water, the antibacterial QACs compounds were quickly released from the materials (AA–QACs or AA–AgNO_3_–QACs), as demonstrated in Fig. 2, and several consecutive immersions led to complete emptying of the nanopores. These results demonstrate that AA materials impregnated with QACs would not be recommended for applications where surfaces are frequently or constantly immersed in a liquid environment (*e.g.*, baths, toilets, taps, *etc.*).

Altogether, our results demonstrate the need to define the appropriate applications and the most suitable and least damaging sanitation protocols for antibacterial materials in order to assess the durability of their antibacterial properties.^116,117^ Along the same line, most international standards are in the process of being updated since they do not currently assess the time and cleaning products' impacts on the antibacterial properties of materials, and they do not consider the conditions of use.

## 4. Conclusions

One of the biggest challenges in the design of antibacterial high-touch surfaces is the sustainable conservation of their properties. The antibacterial properties of materials (inherent material activity or surface modifications) fluctuate depending on the type of bacteria with which they interact, the typical surface wear (*e.g.*, fouling), the conditions of their use (humidity, temperature, dry or liquid environment, *etc.*) and the cleaning and disinfection procedures. These parameters should always be properly characterized and considered when developing and evaluating their antibacterial properties. In this study, nanoporous AA–QACs surfaces demonstrated excellent and rapid antibacterial efficiency on a wide range of Gram-positive and Gram-negative bacteria, independent of the presence of a capsule or antibiotic resistance. Although AA–QACs surfaces were not sporicidal, they nonetheless outperformed copper alloys, considered to be reference antibacterial materials. Furthermore, this high performance in short contact times has been demonstrated under conditions mimicking various surface contamination scenarios: liquid (swab), humid (contaminated gel) and dry (contaminated filter). Importantly, these materials must be used in dry environments (not submerged in liquids) and should be cleaned with QACs-based solutions to preserve their antibacterial properties. Hard-anodized aluminum materials impregnated with QACs constitute a promising innovative strategy to prevent the transmission of pathogenic bacteria linked to the contaminated inert environment.

## Author contributions

JJ and OD performed the experiments. JJ, OD, NF and LCF analyzed the data. JJ, OD GS, XGC, NF and LCF designed the study and experiments. MAG provided the materials. JJ, NF and LCF wrote the manuscript and all authors revised and approved the final version.

## Conflicts of interest

The authors declare that they have no known competing financial interests or personal relationships that could have appeared to influence the work reported in this paper.

## Supplementary Material

RA-011-D1RA07159A-s001
