# Supplementary material for: Rapid antibacterial activity of anodized aluminum-based materials impregnated with quaternary ammonium compounds for high-touch surfaces to limit transmission of pathogenic bacteria
Source: RSC Adv. 2021 Nov 26;11(60):38172–88. doi: 10.1039/d1ra07159a (PMC9044312; doi:10.1039/d1ra07159a)
Supplement: RA-011-D1RA07159A-s001 [file RA-011-D1RA07159A-s001.pdf]

Electronic Supplementary Material (ESI) for RSC Advances.
This journal is © The Royal Society of Chemistry 2021

Electronic Supporting Information (ESI)

Rapid antibacterial activity of anodized aluminum-based materials impregnated with quaternary ammonium compounds for high-touch surfaces to limit transmission of pathogenic bacteria

Jessica Jann [a,b], Olivier Drevelle [a], X. Grant Chen [c], Myriam Auclair Gilbert [d], Gervais Soucy [a], Nathalie Faucheux [*a,b], and Louis Charles Fortier [*e]

[a.] Department of Chemical and Biotechnological Engineering, Faculty of Engineering, Université de Sherbrooke, 2500 boul. de l'Université, Sherbrooke, Québec, J1K 2R1, Canada.

[b.] Clinical Research Center of Centre Hospitalier Universitaire de Sherbrooke, 12e Avenue N, Sherbrooke, Québec, J1H 5N4, Canada

[c.] Department of Applied Science, University of Quebec in Chicoutimi, Saguenay, Quebec, G7H 2B1, Canada

[d.] A3 Surfaces, 1433, Rue de la Manic, Chicoutimi, Québec, G7K 1G8, Canada

[e.] Department of Microbiology and Infectious Diseases, Faculty of Medicine and Health Sciences, Université de Sherbrooke, 3201 rue Jean Mignault, Sherbrooke, Québec, J1E 4K8, Canada.

*Corresponding authors: Prof. Louis-Charles Fortier (Louis-Charles.Fortier@USherbrooke.ca) and Prof. Nathalie Faucheux (Nathalie.Faucheux@USherbrooke.ca)

Contents:

Table S1: Characteristics of bacterial strains used in the present study

Figure S1: Manufacturing steps of A3S materials

Figure S2: Standard curve representing the absorbance (λ: 215 nm) in function of the concentration of QACs contained in the stock solution used for material impregnation

Figure S3: Sporicidal effect of impregnation solutions on *C. difficile* spores

Figure S4: Swab liquid-inoculation assay evaluating the antibacterial activity of different materials on *S. aureus*, *C. difficile* and streptomycin-resistant *S. typhimurium*.

Figure S5: Humid-transfer inoculation assay from contaminated gel evaluating the antibacterial activity of materials

Figure S6: Dry-transfer inoculation assay evaluating the antibacterial activity of materials after contact with a contaminated filter under low humidity conditions

Figure S7: Swab liquid-inoculation assay evaluating the sporicidal activity of the materials on *C.* difficile spores

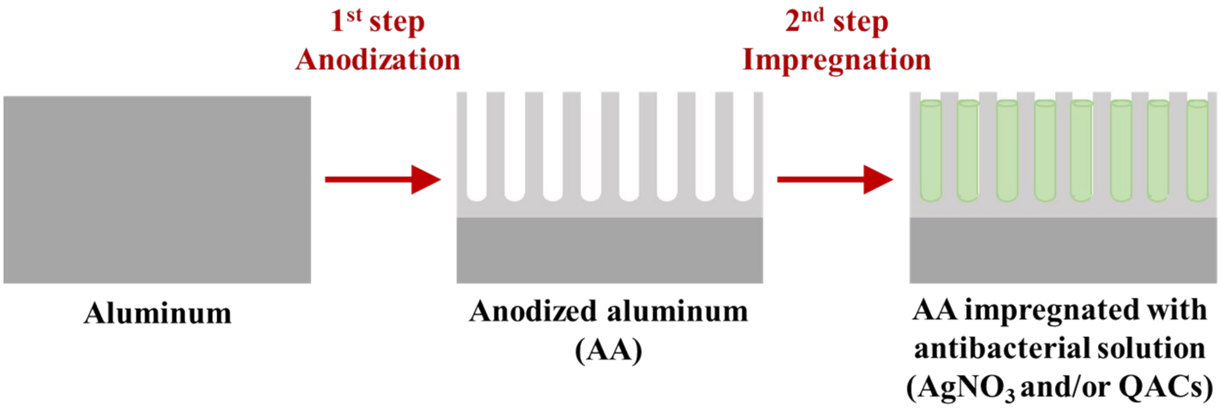

Fig. S1: Manufacturing of A3S materials:
(1) Anodization step: electrolytic process performed in an acid bath allows the formation of nanoporous anodic aluminium oxide, with controlled depth and diameter, on the surface of aluminum sample. **(2)** Impregnation step: antibacterial solution ($AgNO_3$ and/or QACs) is incorporated into the nanopores, followed by a sealing process as final step.

Table S1: Characteristics of bacterial strains used in the present study

Name	# Origin	Gram	Morphology	Cellular dimension (average)	E-test (resistance)	Gene	Culture Medium
Staphylococcus aureus	ATCC # 29213	Gram-positive	Cocci (grape-like clusters)	0.5-1.5 µm diameter	-	-	Mueller Hinton Agar/Broth
Clostridioides difficile	Epidemic strains R20291	Gram-positive	Bacilli	1.0 µm wide by 3.0-4.0 µm long	-	-	Tryptone Yeast extract (TY) Agar/Broth
Enterococcus faecium	Clinical from CHUS #422	Gram-positive	Cocci	0.6-2.5 µm diameter	Vancomycin: > 256 µg/ml	*VanA*	Brain Heart Infusion (BHI) Agar/Broth
Enterococcus faecalis	Clinical from CHUS #55	Gram-positive	Cocci	0.6-2.5 µm diameter	Vancomycin: 8 µg/ml	*VanB*	BHI Agar/Broth
Klebsiella pneumoniae	Clinical from CHUS #455814871	Gram-negative	Capsulated bacilli	0.3-0.8 µm wide by 1.0-3.0 µm long	-	-	Mueller Hinton Agar/Broth
Escherichia coli	ATCC # 29532	Gram-negative	Bacilli	1.0 µm wide by 1.0-2.0 µm long	-	-	Luria-Bertani (LB) Agar/Broth
Salmonella typhimurium	SL1344	Gram-negative	Bacilli	0.7-1.5 µm wide by 2.2-5.0 µm long	Streptomycin: 100 µg/ml	-	LB Agar/Broth + 100 µg/ml Streptomycin

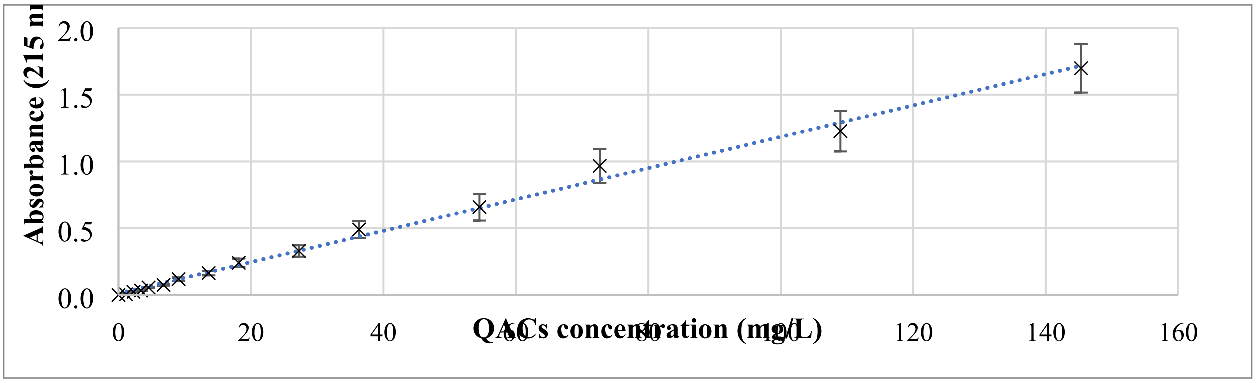

Fig. S2: Standard curve representing the absorbance at a wavelength (λ) of 215 nm in function of the concentration of QACs contained in the stock solution used for material impregnation. The coefficient of determination R^2 = 0.9948, of the standard curve $y = 0.0117x + 0.012$, demonstrates the good reliability of this linear regression model. Results are means ± SEM (n=5; N=15).

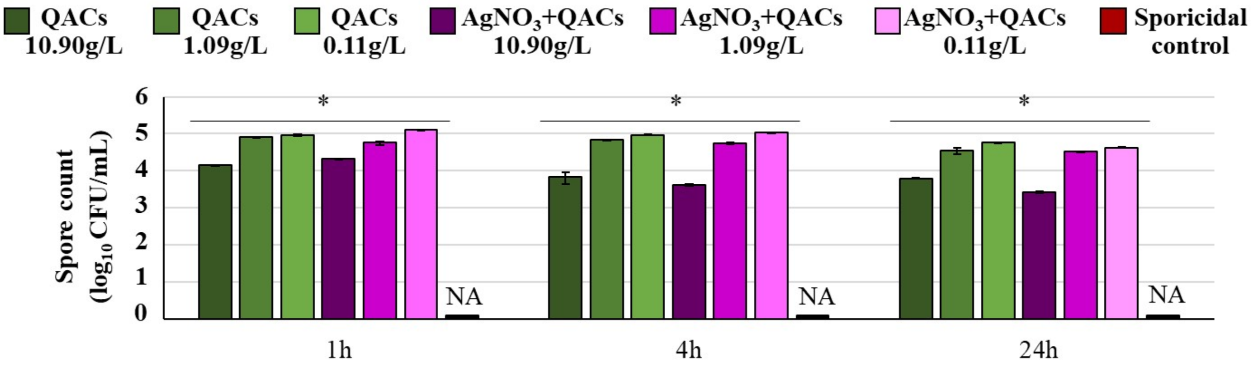

Fig. S3: Sporicidal effect of impregnation solutions (QACs and $AgNO_3$+QACs) on *C. difficile* spores. From an initial inoculum of ≃ 1.6 x 10^5 spores/mL, the number of surviving spores was determined following a contact of 1, 4 and 24 h with impregnation solutions (QACs and $AgNO_3$+QACs) and sporicidal control (1/16 (v/v): Virox^{TM}5/water). Results are means ± SEM (n=4; N=16). Significant effect compared to sporicidal control: *p<0.001; NA: no spore counted.

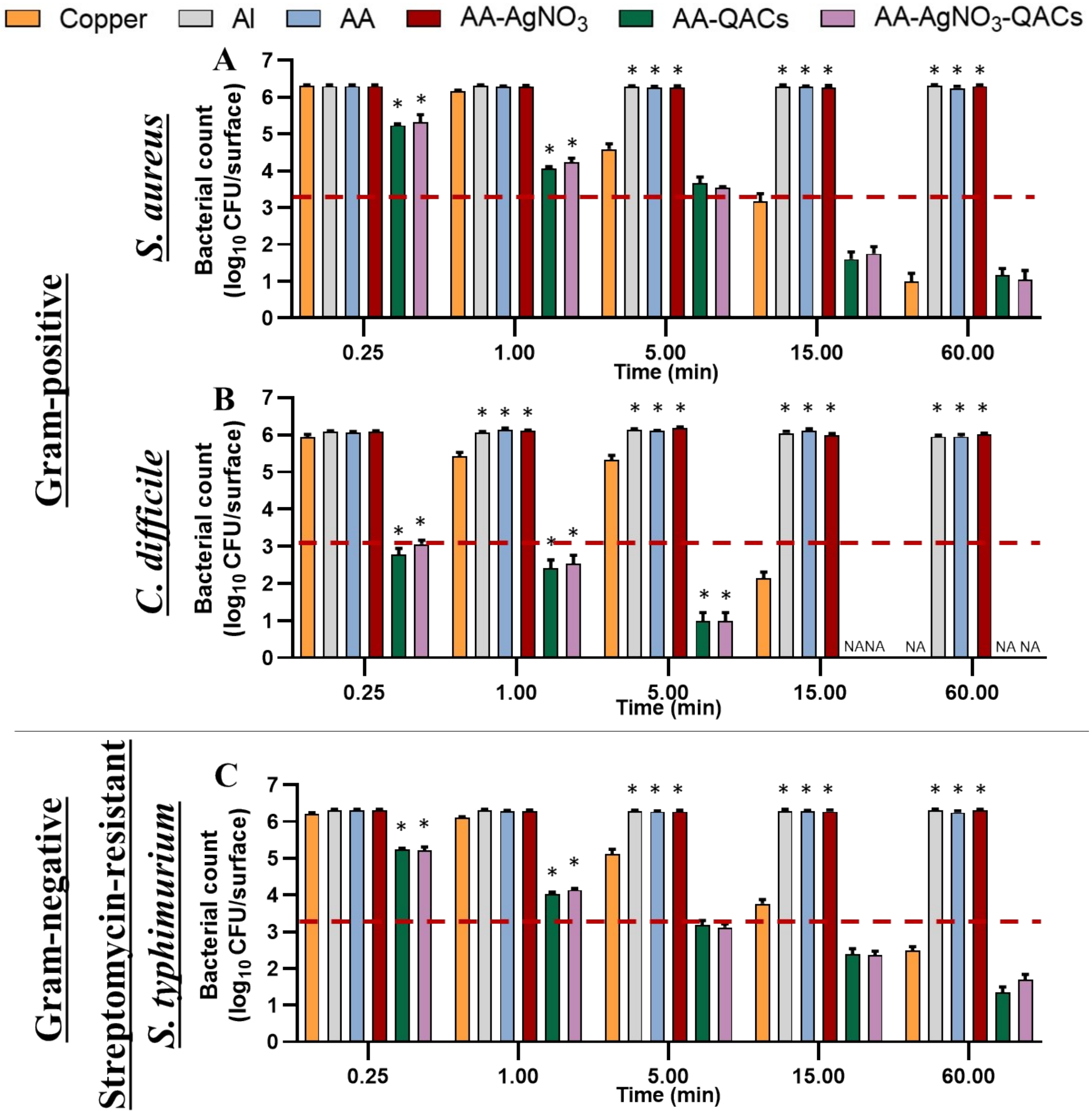

Fig. S4: Swab liquid-inoculation assay evaluating the antibacterial activity of different materials on *S. aureus*, *C. difficile* and streptomycin-resistant *S. typhimurium*. The reference of 99.9% antibacterial activity is indicated by the dotted red line. Results are means ± SEM (n=2; N=8). Statistical effect compared to copper: * $p < 0.001$; NA: no bacteria counted.

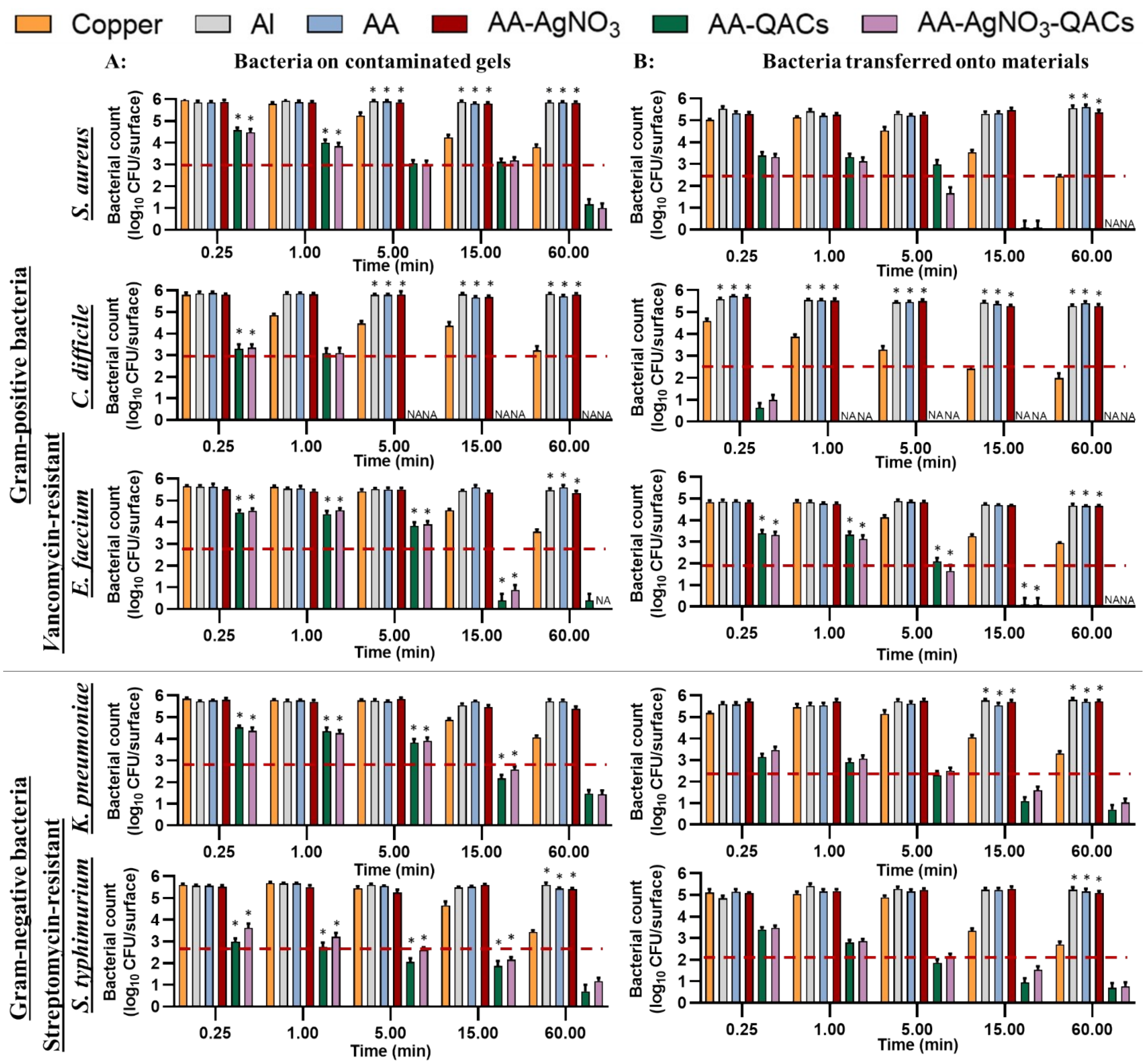

Fig. S5: Humid-transfer inoculation assay from contaminated gel evaluating the antibacterial activity of materials. The graph shows total counts of bacteria that survived **(A)** on the gel or **(B)** after transfer from the gel to the different materials. The reference of 99.9% antibacterial activity is indicated by the dotted red line. Results are means ± SEM (n=2; N=8). Statistical significance when compared to copper: * p<0.001; NA: no bacteria counted.

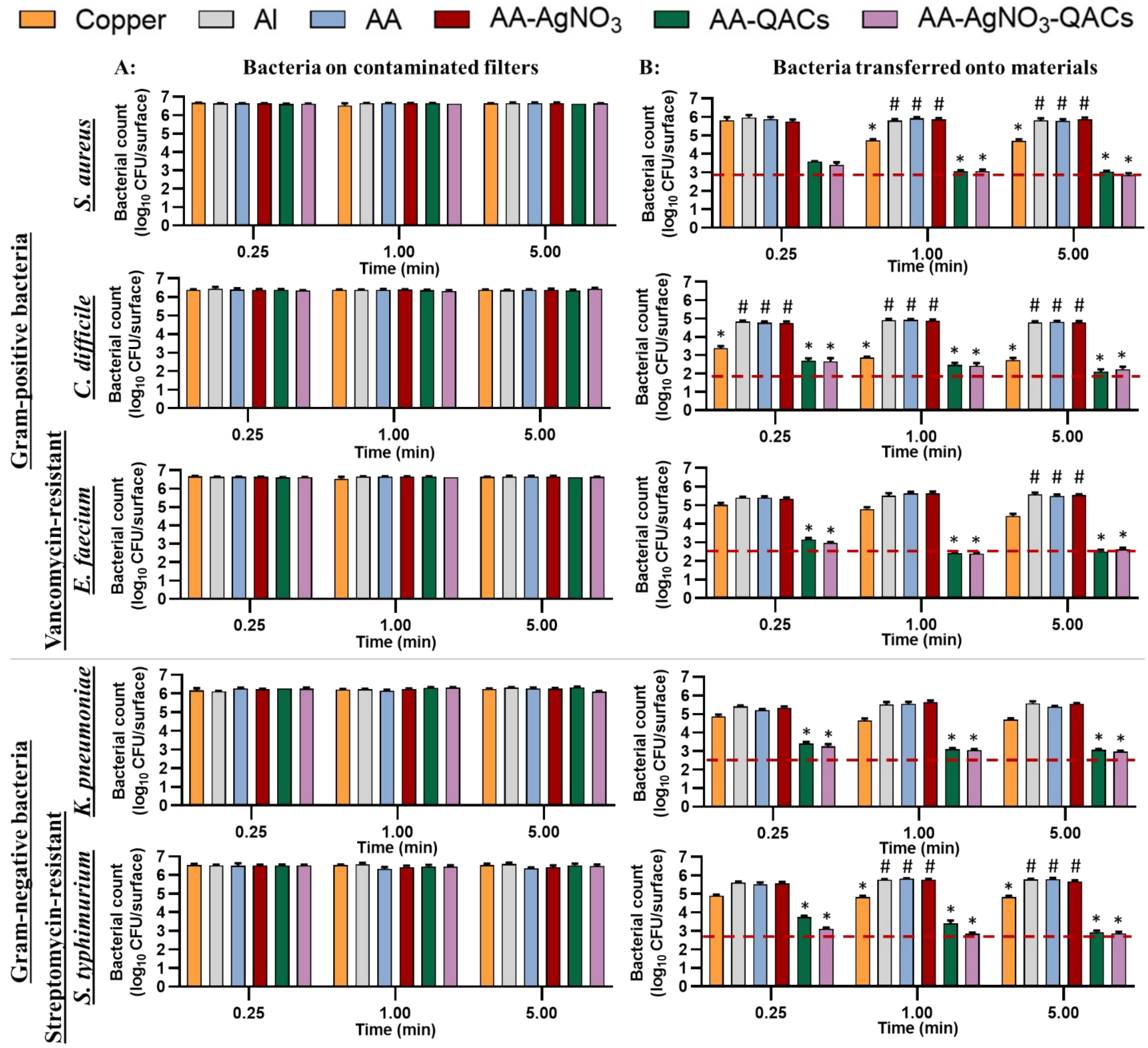

Fig. S6: Dry-transfer inoculation assay evaluating the antibacterial activity of materials after contact with a contaminated filter under low humidity conditions. Counts in log_{10} CFU/surface indicate the number of bacteria that survived on **(A)** the contaminated filters and **(B)** the materials, after transfer from the filter. Results are means ± SEM (n=2; N=8). Statistical effect compared to AA: *p<0.001 and to copper: #p<0.001.

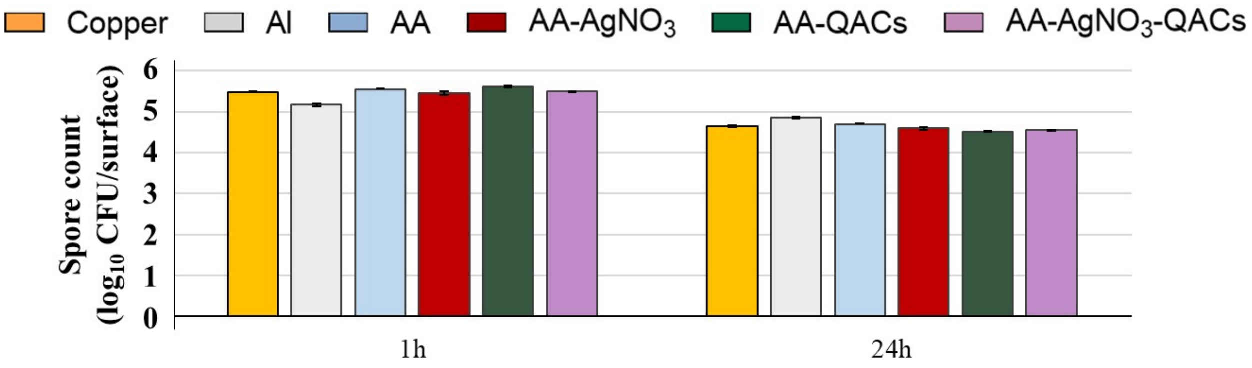

Fig. S7: Swab liquid-inoculation assay evaluating the sporicidal activity of the materials on *C. difficile* spores following a contact of 1 and 24 h. Results are means ± SEM (n=3; N=12). No statistical effect was observed.
